# Dataset on radioactivity measurement of Beryllium mining field in Ifelodun and gold mining field in Moro, Kwara State, North-central Nigeria

**DOI:** 10.1016/j.dib.2020.105888

**Published:** 2020-06-18

**Authors:** Muyiwa Michael Orosun, Kayode John Oyewumi, Mojisola Rachael Usikalu, Charity Adaeze Onumejor

**Affiliations:** a Department of Physics, University of Ilorin, Ilorin, Kwara State, Nigeria; b Department of Physics, Covenant University, Ogun State, Nigeria

**Keywords:** Radioactivity, Beryllium, Gold, Super-Spec RS-125, Kwara, Nigeria

## Abstract

This work contains dataset of measured activity concentrations of *^40^K, ^238^U, ^232^Th* and gamma doses at 1 m above the ground level over Beryllium and Gold mining fields in Ifelodun and Moro respectively, Kwara State, North-central Nigeria. A well calibrated Super-Spec (RS-125) gamma spectrometer was used to carry out these measurements. Measurements were carried out manually in 72 randomly selected sample points. Statistical analyses of the data were explored to infer potential statistical relationships. The obtained dataset is presented for further assessment that can offer insights into the safety state of Ifelodu, Moro and their environs from radiation protection point of view. The data in this study could serve as a substantial baseline radiological data of the region for future monitoring and epidemiology researches.

**Specifications Table**

**Table:** 

Subject	Physics and Astronomy
Specific subject area	Radiation
Type of data	Table; Figure
How data wereacquired	Super SPEC RS-125 spectrometer with large 2.0 × 2.0 NaI crystal was used to the radioactivity measurements. The coordinate and elevation were determined using a global positioning system (GPSMAP78)
Data format	Raw analysed
Parameters for data collection	The measurement of the activity concentration of the radionuclides was carried out at about 1 metre above the topsoil.
Description of data collection	Four (4) readings were recorded at each data point at the interval of 120 s. 72 sample points were recorded in total to cover the two mining fields. The field was divided into grids of approximately equal size, with each box representing a data collection point. At each of these samples location, the coordinate and elevation were determined using a global positioning system (GPSMAP78)
Data source location	Ifelodun and Moro Local Government Area (L.G.A.), Kwara State, North-central Nigeria. The study area for the data acquisition is bounded by longitude 4.759166° E and latitude 8.435183°N and longitude 4.460320°E and latitude 8.965820° N
Data accessibility	All the data are in this data article

**Value of the data**

• The data in this work are useful because they provide useable information on the concentrations level of activity concentrations of *^40^K, ^238^U, ^232^Th* and gamma dose, which could be used to evaluate the degree of ionizing radiation exposure and probable radiological impacts within Ifelodun, Moro and their environs.
• The dataset could be used to compute the radiological impact parameters such as the absorbed dose rate, annual effective dose, external and internal radiation hazard indices and excess life time cancer risk due to the concentration of natural radionuclides in the samples. Radiological risks assessment and health implications could also be estimated by comparing them with the international recommended limits.
• The radioactivity measurements can be repeated in mining sites in the neighboring locations such as Asa, Ekiti, Patigi and other Local Government Areas (LGA). This radiometric survey can also be extended to cover all the mining sites in the whole of Kwara state.
• The dataset in this work is useful for educational purposes in health and radiation Physics i.e. in applications of gamma spectrometry methods and environmental radioactivity.The dataset could also be used by the Nigerian Environmental Protection Agency (NEPA) and other regulatory bodies for monitoring and assessment of radiological implication of mining activities so as to implement specific statutory requirements and laws to regulate the mining activities in the country. Similar works can be found in [1], [2], [3], [4], [5], [6].

## 1 Data description

The dataset covers the measured concentration levels of activity concentrations of *^40^K, ^238^U, ^232^Th* and gamma dose rate for Beryllium and Gold mining locations in Ifelodun and Moro LGAs respectively, in Kwara State, North-central Nigeria. The measured activity concentration of these natural radionuclides considered and their geographical coordinatesis given in Tables 1 and 3. Additionally, descriptive statistical analyses were carried out on the raw dataset to comprehend the statistical distribution of the measurements. The statistical summary of the dataset is presented in Tables 2 and 4. The correlation analysis was conducted as shown in Tables 5 and 6 to study the relationship between these measured radionuclides and the gamma dose rate.

**Table 1:** Measured activity concentrations of ^4^*^0^K, ^238^U, ^232^Th* and the dose rates (*DR*) from Beryllium mining field in Ifelodun LGA.

CODE	Longitude °E	Latitude °N	*DR* (nGyh^−1^)	*^40^K* (Bqkg^−1^)	*^238^U* (Bqkg^−1^)	*^232^Th* (Bqkg^−1^)
IFES1	4.759167	8.435183	51.10	626.00	46.93	6.50
IFES2	4.759133	8.435167	53.40	876.40	1.24	25.17
IFES3	4.759117	8.435150	63.90	813.80	17.29	34.51
IFES4	4.758450	8.435100	49.50	719.90	32.11	8.53
IFES5	4.759100	8.435083	50.60	813.80	1.24	24.77
IFES6	4.759100	8.433667	49.80	500.80	46.93	7.71
IFES7	4.758483	8.435033	26.30	281.70	9.88	15.83
IFES8	4.759167	8.435050	37.40	438.20	13.59	19.89
IFES9	4.759183	8.435100	45.80	344.30	8.65	41.01
IFES10	4.759167	8.435133	60.70	845.10	1.24	38.98
IFES11	4.759200	8.435167	40.60	594.70	1.24	24.36
IFES12	4.759217	8.435200	52.20	845.10	18.53	9.34
IFES13	4.759233	8.435183	27.90	344.30	17.29	8.53
IFES14	4.759200	8.435183	22.00	187.80	1.24	21.11
IFES15	4.759200	8.435150	26.20	344.30	1.24	32.07
IFES16	4.759200	8.435133	45.10	187.80	46.93	25.58
IFES17	4.759200	8.435117	41.50	187.80	44.46	21.52
IFES18	4.759183	8.435100	22.70	219.10	21.00	5.68
IFES19	4.759200	8.435083	19.10	250.40	3.71	10.15
IFES20	4.759217	8.435117	34.60	250.40	6.18	32.89
IFES21	4.759233	8.435133	43.00	156.50	34.58	32.48
IFES22	4.759233	8.435133	31.60	93.90	30.88	20.71
IFES23	4.759233	8.435167	28.90	125.20	40.76	8.93
IFES24	4.759250	8.435183	29.80	156.50	40.76	8.12
IFES25	4.759083	8.435200	36.80	500.80	21.00	10.15
IFES26	4.759083	8.435183	58.00	1001.60	28.41	5.68
IFES27	4.759050	8.435167	51.70	375.60	8.65	41.01
IFES28	4.759050	8.435150	26.20	375.60	4.94	12.99
IFES29	4.759050	8.435133	43.00	438.20	18.53	25.17
IFES30	4.759033	8.435133	38.00	375.60	21.00	19.49
IFES31	4.759000	8.435133	29.80	281.70	6.18	22.33
IFES32	4.759017	8.435167	34.90	375.60	1.24	29.64
IFES33	4.759017	8.435200	24.10	219.10	6.18	17.46
IFES34	4.759033	8.435217	40.70	187.80	24.70	32.48
IFES35	4.759033	8.435233	48.90	187.80	65.46	19.49
IFES36	4.759067	8.435250	37.40	344.30	19.76	21.92

**Table 2:** Statistical summary of the measured activity concentrations of the natural radionuclides and the gamma dose rate of Beryllium mining field in Ifelodun LGA.

	*DR* (nGyh^−1^)	*^40^K* (Bqkg^−1^)	*^238^U* (Bqkg^−1^)	*^232^Th* (Bqkg^−1^)
Min	19.10	93.90	1.24	5.68
Max	63.90	1001.60	65.46	41.01
Range	44.80	907.70	64.22	35.32
Sum	1423.20	14867.50	713.83	742.17
Mean	39.53	412.99	19.83	20.62
Median	40.60	375.60	18.53	21.11
Mode	37.40	187.80	1.24	25.17
SD	11.80	250.83	17.09	10.62
CV	29.86	60.73	86.20	51.51
Skewness	0.15	0.89	0.79	0.27
Kurtosis	−0.88	−0.33	−0.14	−0.92

**Table 3:** Measured activity concentrations of ^4^*^0^K, ^238^U, ^232^Th* and the dose rates (*DR*) from Gold mining field in Moro LGA.

CODE	Latitude °N	Longitude °E	*DR* (nGyh^−1^)	*^40^K* (Bqkg^−1^)	*^238^U* (Bqkg^−1^)	*^232^Th* (Bqkg^−1^)
MORS1	8.965820	4.460320	71.80	594.70	45.70	41.01
MORS2	8.965820	4.460280	80.00	813.80	45.70	38.98
MORS3	8.965850	4.460280	77.00	751.20	65.46	24.36
MORS4	8.965880	4.460250	85.50	626.00	72.87	41.01
MORS5	8.965880	4.460250	88.50	657.30	60.52	11.37
MORS6	8.965900	4.460220	85.50	751.20	28.41	62.12
MORS7	8.965930	4.460230	85.10	626.00	48.17	59.68
MORS8	8.965900	4.460270	74.10	719.90	30.88	46.28
MORS9	8.965870	4.460280	81.80	594.70	51.87	51.97
MORS10	8.965870	4.460300	71.10	532.10	28.41	52.78
MORS11	8.965850	4.460330	61.90	532.10	8.65	54.40
MORS12	8.965830	4.460330	89.00	970.30	16.06	73.89
MORS13	8.965850	4.460370	73.70	594.70	23.47	58.06
MORS14	8.965870	4.460350	88.20	876.40	54.34	41.01
MORS15	8.965880	4.460330	70.30	563.40	43.23	41.82
MORS16	8.965920	4.460320	78.00	406.90	97.57	28.42
MORS17	8.965930	4.460280	56.30	532.10	25.94	34.10
MORS18	8.965930	4.460270	77.60	406.90	79.04	39.38
MORS19	8.965750	4.460300	69.60	500.80	76.57	22.33
MORS20	8.965770	4.460280	73.10	563.40	56.81	37.35
MORS21	8.965820	4.460260	83.10	876.40	55.58	33.70
MORS22	8.965800	4.460250	77.10	939.00	4.94	54.81
MORS23	8.965800	4.460220	80.20	719.90	12.35	66.58
MORS24	8.965800	4.460160	73.90	876.40	3.71	53.19
MORS25	8.965800	4.460180	74.40	626.00	39.52	45.88
MORS26	8.965780	4.460180	75.80	688.60	43.23	41.82
MORS27	8.965770	4.460230	81.10	907.70	11.12	58.46
MORS28	8.965750	4.460230	68.60	563.40	70.40	21.11
MORS29	8.965730	4.460270	78.80	626.00	61.75	37.35
MORS30	8.965730	4.460300	66.80	438.20	24.70	58.06
MORS31	8.965700	4.460280	87.90	719.90	55.58	49.53
MORS32	8.965720	4.460250	70.80	469.50	29.64	58.06
MORS33	8.965730	4.460230	82.50	438.20	77.81	44.66
MORS34	8.965730	4.460220	76.50	563.40	22.23	66.18
MORS35	8.965730	4.460200	90.30	657.30	65.46	42.22
MORS36	8.965730	4.460160	70.20	594.70	27.17	49.94

**Table 4:** Statistical summary of the measured activity concentrations of the natural radionuclides and the gamma dose rate of Beryllium mining field in Ifelodun LGA.

	*DR* (nGyh^−1^)	*^40^K* (Bqkg^−1^)	*^238^U* (Bqkg^−1^)	*^232^Th* (Bqkg^−1^)
Min	56.30	406.90	3.71	11.37
Max	90.30	970.30	97.57	73.89
Range	34.00	563.40	93.86	62.52
Sum	2776.10	23318.50	1564.75	1641.86
Mean	77.11	647.74	43.47	45.61
Median	77.05	626.00	44.46	45.27
Mode	85.50	594.70	45.70	41.01
SD	7.87	152.94	23.89	13.91
CV	10.21	23.61	54.97	30.49
Skew	−0.36	0.48	0.16	−0.33
Kurt	0.11	−0.50	−0.74	0.01

**Table 5:** Pearson's correlation matrix showing the relationship between the measured radionuclides and the gamma dose rate at Beryllium mine field in Ifelodun LGA.

	*DR* (nGyh^−1^)	*^40^K* (Bqkg^−1^)	*^238^U* (Bqkg^−1^)	*^232^Th* (Bqkg^−1^)
***DR* (nGyh^−1^)**	1.0000			
***^40^K* (Bqkg^−1^)**	0.7210	1.0000		
***^238^U* (Bqkg^−1^)**	0.2302	0.2366	1.0000	
***^232^Th* (Bqkg^−1^)**	0.2916	0.0361	0.3833	1.0000

**Table 6:** Pearson's correlation matrix showing the relationship between the measured radionuclides and the gamma dose rate at Gold mine field in Moro LGA.

	*DR* (nGyh^−1^)	*^40^K* (Bqkg^−1^)	*^238^U* (Bqkg^−1^)	*^232^Th* (Bqkg^−1^)
***DR* (nGyh^−1^)**	1.0000			
***^40^K* (Bqkg^−1^)**	0.4761	1.0000		
***^238^U* (Bqkg^−1^)**	0.2788	0.4213	1.0000	
***^232^Th* (Bqkg^−1^)**	0.0889	0.2296	0.7153	1.0000

The unceasing release of mining waste and tailings into the biosphere from mining activities has been reported to bring about building-up of radionuclides in soil, air, water and the food chain. According to [7–9] report, the recommended values for general populace for exposure to *^40^K, ^238^U, ^232^Th* and *DR* are given as 420.00, 32.00, 45.00 Bq kg^−1^and 59.00 nGy h^−1^, respectively. When the levels of the activity concentrations of these aforementioned radionuclides and the gamma doserate exceeds these minimum permissible limits, it can cause severe health effects like cancer and other radiation health effects, damaging critical organs of the body which could even lead to death [3–5,[9], [10], [11]].

## 2 Experimental design, materials, and methods

### 2.1 Study area

**Fig. 1:**
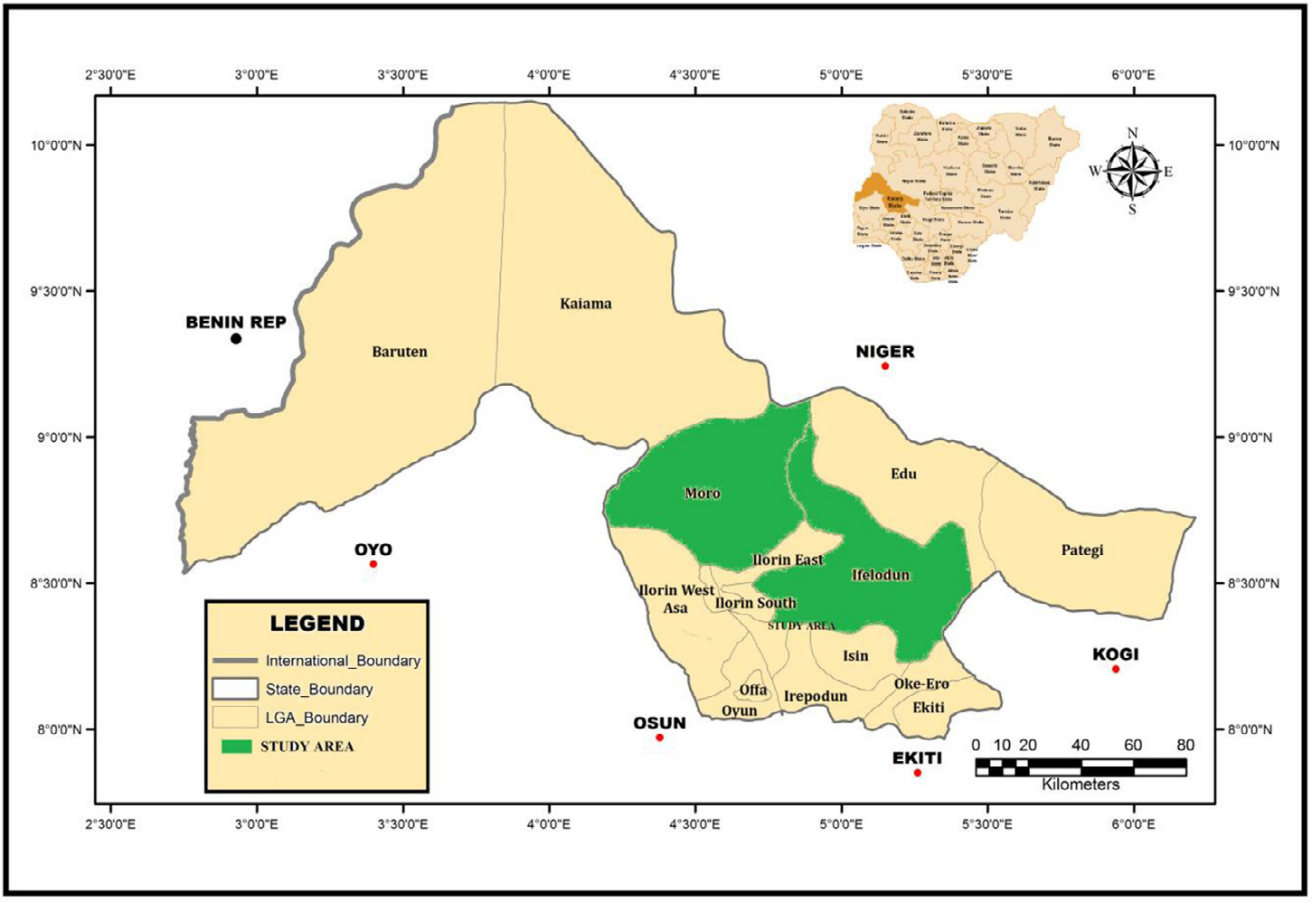
Geological map of Nigeria showing the study areas.

The study areas are in Ifelodun and Moro LGAs of Kwara state, Nigeria. It is situated between latitudes 8°20′ N and 8°50′ N and Longitudes 4°25′ E and 4°65′E (Fig. 1). Kwara is located in the North-central part of Nigeria with a tropical wet and dry climate with average yearly rainfall of about 1200 mm [12]. Its mean annual temperature is about 26.2 °C; which peaks at about 30 °C in the month of March. Wet season is generally experienced between April and October and dry season between November and March.

For the geology of the study area, a large part of Kwarais underlain by basement complex rock. The soils are formed from basement complex rocks (metamorphic and igneous rocks) which is about 95%. The metamorphic rocks consist of biotitegnesiss, banded gnesiss, quartzite augitegnesiss and granitic gnesiss. The intrusive rock comprises of pegmatite and vein quartz [12], [13], [14], [15]. The assortment of basement complex rocks brings about large number of ferruginous groups of soils. Therefore, lateritic soil type (generally deep red in colour with high clay content) is the major type of soil in Ilorin. Detail geology of Ilorin can be found in [12], [13], [14], [15].

### 2.2 Field survey

Measurements of activity concentrations of ^40^K, ^232^Th, ^238^U and the radiation dose exposures were carried*in-situ*using Super SPEC RS-125 spectrometer with large 2.0 × 2.0 NaIcrystal. The measurement of the activity concentration of the natural radionuclides and the gamma dose rate was carried out at about 1 metre above the ground [2,3,6]. The Super Spec (RS-125) gamma spectrometer is a portable handheld radiation detector with high precision and probable error of about 5%. It offersbetter integrated design with largedetector,decent sensitivity and it is easy to use. The model RS-125 super-spec is manufactured by Canadian Geophysical Institute, Canada. It comes with a big data storage which allows measurement of multiple readings. The Super Spec spectrometer was calibrated in accordance with Canadian Geophysical Institute. The calibration was carried out on 1 × 1 m test pads, which uses 5 min spectra accumulation on uranium, thorium and potassium pads and 10 min accumulation on the Background pad. It uses sodiumiodide (NaI) crystal which is doped with thallium [Tl] as activator. The instrument's energy range is from 30 to 3000 keV, which is sufficient to detect most of the radiation giving off from the terrestrial sources (i.e. ^214^Bi (609.31 and 1764.49 keV) gamma rays to determine ^238^U, ^212^Pb (238.63 keV), ^208^Tl (583.19 keV) and ^228^Ac (911.21 keV) gamma rays to determine ^232^Th and the photopeaks of ^40^K which occours in the background spectrum at 1460.83 keV). The total count of 120 s per assay was employed for best accuracy as stated in Radiation Solutions Inc [2,3,16]. The assay mode of the instrument gives the activity concentration of ^40^K in percentage (%), ^238^U and ^232^Th in part per million (ppm). The data was converted to the conventional unit *Bqkg^−1^*using conversion factors given by [8,9,17,18].

In this work, four (4) readings were recorded at each data point at the interval of 120 s. 72 sample points were recorded in total to cover the two mining fields. The field was divided into grids of approximately equal size, with each box representing a data collection point. At each of these samples location, the coordinate and elevation were determined using a global positioning system (GPSMAP78). More details about the instrument can be found in earlier works where this Super SPEC RS-125spectrometer was used [2,3,6,18,19].

### 2.3 Descriptive statistics

The detailed statistical descriptions of the results of the *in-situ*gamma spectrometry measurements using Super-Spec RS125 is presented in Tables 2 and 4. It shows their estimated minimum, maximum, range, sum, mean, median, mode, standard deviation (SD), Skewness and Kurtosis. The measured values for all the parameters (i.e. *^238^U, ^232^Th, ^40^K* and *DR*) were slightly skewed (i.e. the distribution is approximately symmetric) since most of the measure of the asymmetry of their probability distribution about their means is in the range of −1 and +1 [20].

**Fig. 2:**
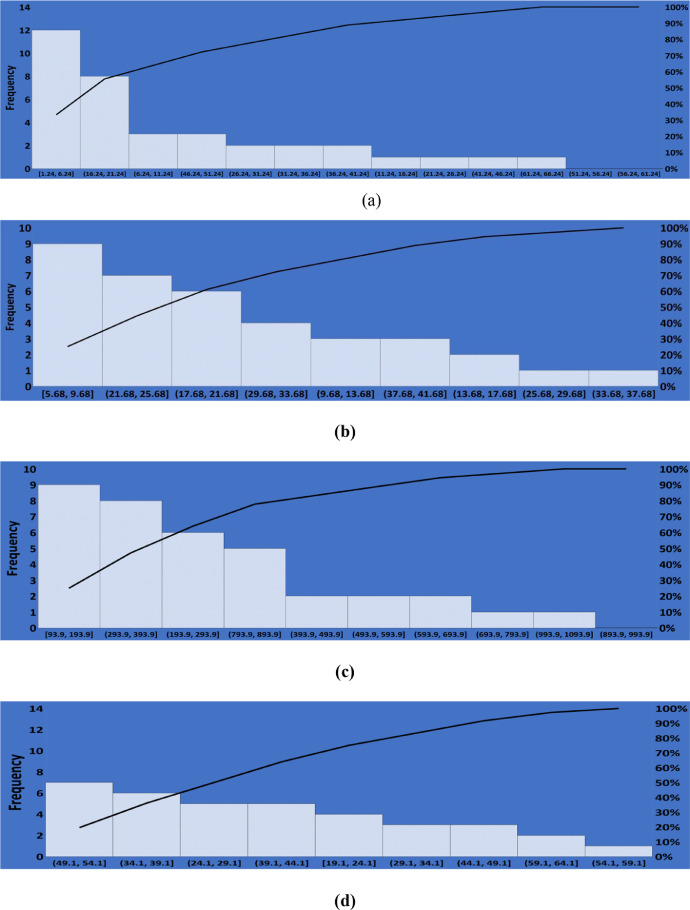
Pareto Chart (a) *^238^U*, (b) *^234^Th*, (c) *^40^K* and (d) Dose Rate (*DR*).

The calculation of coefficient of variation (CV) also reveals the variability in the distribution of the measured activity concentrations of *^238^U, ^232^Th,^40^K* and the radiation dose rate in the study area. CV ≤ 20% indicates little variability, 20 < CV ≤ 50% implies moderate variability, while 50% < CV ≤ 100% indicates high variability and CV value greater than 100% is regarded as exceptionally high variability [12,21].The Pareto chart was plotedto showthemost significant factor (set) in the dataset ofeachmeasuredradiogicalparameter. These chartsareshown in Fig. 2(a)–(d).

### 2.4 Correlation analyses

The correlation analysis between the measured activity concentrations of the natural radionuclides and the radiation dose rate was conducted using Pearson techniquesto further investigate theirdegreeofstrengthandnatureof the relationship. The results are given in Tables 5 and 6. The results were classified according to the correlation coefficient R [12, 22], as follows:

0.8 ≤ |R| ≤ 1 suggests a strong correlation;
0.5 ≤ |R| ≤ 0.8 suggests a significant correlation;
0.3 ≤ |R| ≤ 0.5 suggests a weak correlation; and
|R| < 0.3 suggests an insignificant correlation.

## Declaration of Competing Interest

The authors declare that they have no known competing financial interests or personal relationships which have, or could be perceived to have influenced the work reported in this article.

## Appendix Supplementary materials

Image, application 1
